# Electrochemical Activation of Atomic-Layer-Deposited Nickel Oxide for Water Oxidation

**DOI:** 10.1021/acs.jpcc.3c05002

**Published:** 2023-11-08

**Authors:** Sina Haghverdi Khamene, Cristian van Helvoirt, Mihalis N. Tsampas, Mariadriana Creatore

**Affiliations:** † Department of Applied Physics and Science Education, Eindhoven University of Technology, Eindhoven 5600 MB, The Netherlands; ‡ DIFFER—Dutch Institute For Fundamental Energy Research, Eindhoven 5612 AJ, The Netherlands; § Eindhoven Institute for Renewable Energy Systems (EIRES), Eindhoven 5600 MB, The Netherlands

## Abstract

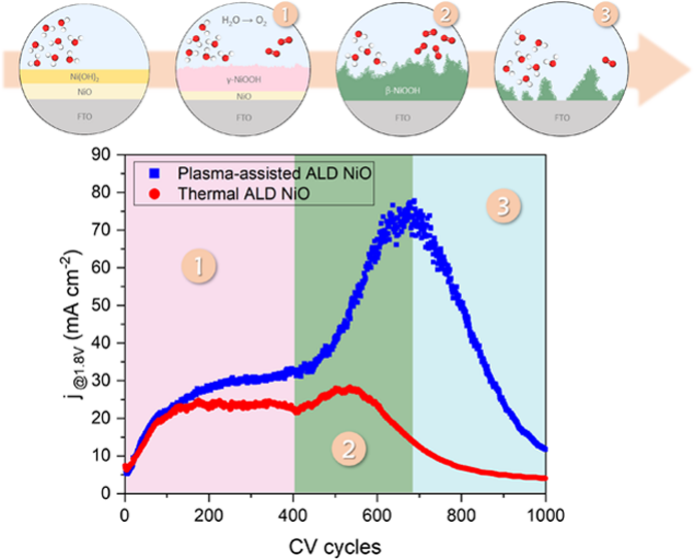

NiO-based electrocatalysts, known for their high activity, stability, and low cost in alkaline media, are recognized as promising candidates for the oxygen evolution reaction (OER). In parallel, atomic layer deposition (ALD) is actively researched for its ability to provide precise control over the synthesis of ultrathin electrocatalytic films, including film thickness, conformality, and chemical composition. This study examines how NiO bulk and surface properties affect the electrocatalytic performance for the OER while focusing on the prolonged electrochemical activation process. Two ALD methods, namely, plasma-assisted and thermal ALD, are employed as tools to deposit NiO films. Cyclic voltammetry analysis of ∼10 nm films in 1.0 M KOH solution reveals a multistep electrochemical activation process accompanied by phase transformation and delamination of activated nanostructures. The plasma-assisted ALD NiO film exhibits three times higher current density at 1.8 V vs RHE than its thermal ALD counterpart due to enhanced β-NiOOH formation during activation, thereby improving the OER activity. Additionally, the rougher surface formed during activation enhanced the overall catalytic activity of the films. The goal is to unravel the relationship between material properties and the performance of the resulting OER, specifically focusing on how the design of the material by ALD can lead to the enhancement of its electrocatalytic performance.

## Introduction

The use of fossil fuels results in the emission of greenhouse gases, which contribute to global warming, climate change, and several related environmental harms.^1,2^ As a result, the energy transition from fossil-based fuels toward renewable energy sources calls for rapid and urgent measures as well as promotes more research efforts in the quest for efficient and cost-effective approaches.^3^ Among the potential alternatives to nonrenewable energy sources, hydrogen is regarded as a key component in the transition to a sustainable energy supply and the primary solution to store wind and solar energy on a massive scale, in addition to the production of electricity.^4,5^ The production of H_2_ via water electrolysis, in which water is split into H_2_ and O_2_, is a key technology to the implementation of a sustainable hydrogen economy and is one of the most prominent CO_2_-free approaches.^6−8^ Nevertheless, the cost and efficiency of water electrolysis are closely tied to the electrocatalyst employed for the oxygen evolution reaction (OER), which is the rate-determining step of the process.^9,10^ The state-of-the-art electrocatalysts for OER based on precious metals suffer from high costs and scarce supply, making them less desirable for industrial and commercial applications.^11^ Accordingly, the development of cost-effective and efficient ion–response ratios of the OER electrocatalysts is of critical importance.

**Figure 1:**
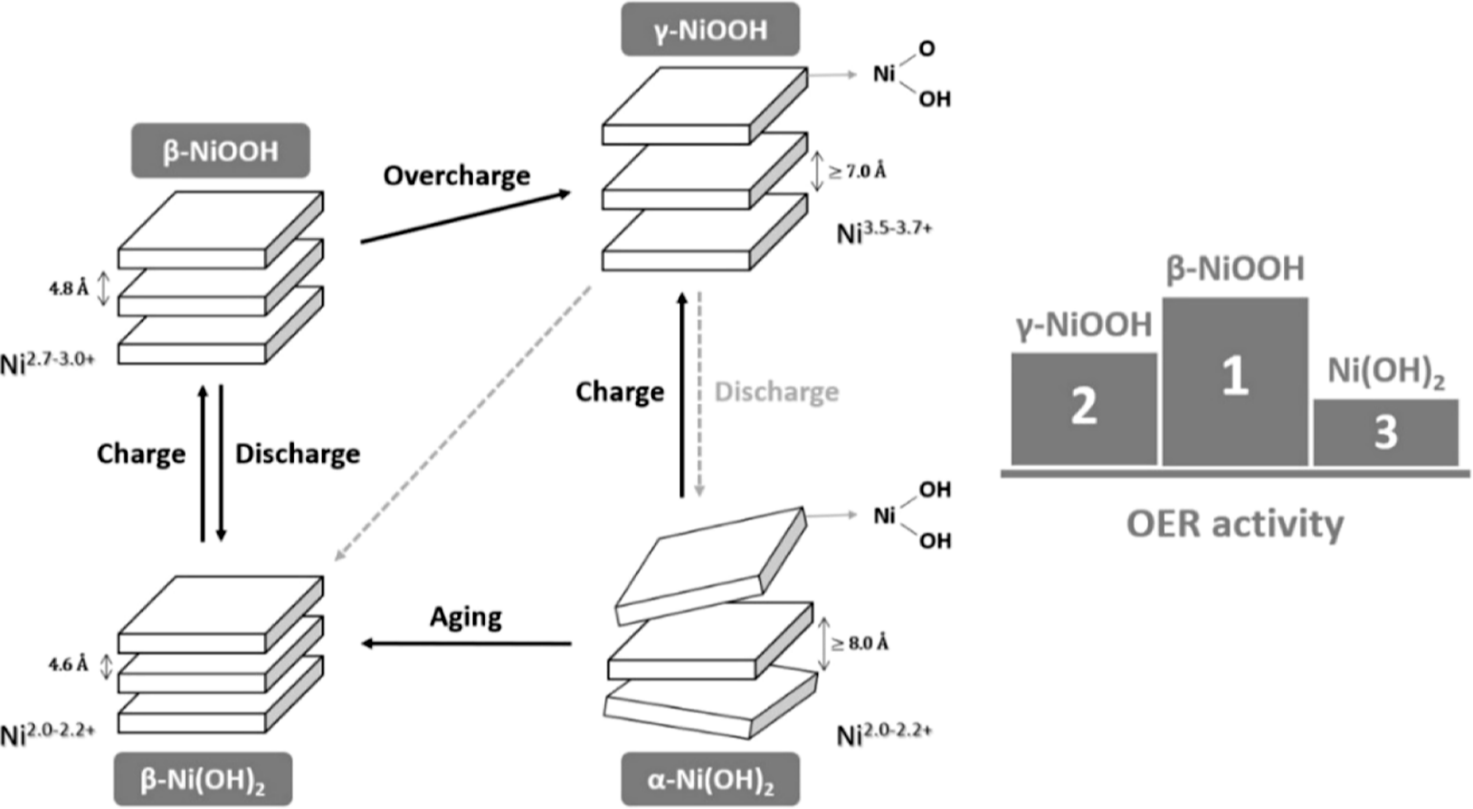
Visualization of the Bode diagram of the Ni(OH)_2_ to NiOOH redox transformation in an alkaline solution.^21,31,34^

Nickel oxide (NiO)-based electrocatalysts have been explored over many years as prospective candidates due to their remarkable activity and stability for the OER in alkaline media.^12−16^ In this regard, investigations into the OER mechanism and structural modifications of NiO-based catalysts throughout the reaction have been the focus of several studies.^17−20^ The Bode model^21^ is a theoretical framework that provides a comprehensive understanding of the OER mechanism and describes the phase transformation that occurs in NiO-based electrocatalysts during OER (Figure 1). It has been established that NiO is converted into Ni(OH)_2_ upon immersion in an alkaline solution due to the presence of hydroxyl ions in the solution.^22^ The model proposes that hydrous α-Ni(OH)_2_ is the initial phase formed during the electrochemical activation of NiO, followed by conversion into γ-NiOOH when a positive potential is applied. Upon aging during the electrochemical activation process, the initial phase α-Ni(OH)_2_ undergoes a transformation into anhydrous β-Ni(OH)_2_. As a result, enhancing the working potential further drives the formation of β-NiOOH.^23−25^ The oxidation state of nickel in the γ-NiOOH phase is larger than that in NiO, i.e., up to 3.5–3.7. However, this phase is known to transform into the β-NiOOH phase with aging, which features a lower oxidation state of 2.7–3.0.^26^ It has been indicated that the β-Ni(OH)_2_/β-NiOOH redox couple is highly advantageous for the OER, as β-NiOOH has a superior performance compared to other phases due to its higher stability and more active sites for catalyzing the OER process.^27−29^ Despite the ongoing debate, the precise mechanism behind the phase transformation during the electrochemical activation process has yet to be fully understood. The process involves the interplay of various physical and chemical phenomena, including the charge transfer, ion diffusion, and structural changes within the material. These complex interactions pose a challenge in precisely determining the specific mechanism underlying the observed phase transformation. Studies have shown that NiO-based catalysts exhibit low activity and stability unless Fe is present, with the gradual introduction of Fe over time stabilizing and crystallizing γ-NiOOH into the β phase, enhancing the catalyst performance. However, the β-phase transforms into γ-NiOOH under overcharging conditions in the absence of Fe.^20,30−33^ Given the potential of the β-Ni(OH)_2_/β-NiOOH redox couple in the OER and the need to control and regulate the phase transformation during the activation process of NiO-based electrocatalysts, developing thin-film model systems is essential to investigate and improve the activation process.

Atomic layer deposition (ALD) is a promising technique for developing electrocatalysts, and it has garnered major attention.^35−37^ ALD enables precise control over the growth of thin films and results in conformal and uniform coatings on various substrates.^38,39^ This capability extends to the deposition of complex 3D structures, making ALD a valuable tool in electrocatalysis. This feature not only increases the concentration of active sites for catalytic reactions but also promotes efficient mass transport of reactants.^40^ In addition, the potential of ALD to tune the chemical composition and thickness of films enables the improvement of their performance and stability for OER.^41−43^ Accordingly, the method has gained significant attention in the development of electrocatalysts for OER.^44−51^ In previous work,^52^ we have shown that ALD enables digital control over the stoichiometry of cobalt phosphate films and that this approach leads to a deeper understanding of their activation mechanism and performance. Moreover, Mattelaer et al.^53^ demonstrated the feasibility of producing active manganese oxide catalysts through ALD. These ALD-deposited films serve as efficient OER catalysts for integrated solar hydrogen devices in alkaline conditions, eliminating the need for postdeposition oxidative treatment and highlighting the significance of precisely controlling the film’s oxidation state to optimize catalyst properties. Additionally, Nardi et al.^54^ have investigated ALD NiO in OER, using Ni(Cp)_2_ as the reactant in combination with O_3_ as a coreactant throughout the process. Their study evaluated the electrocatalytic activity of NiO films in the presence of varying concentrations of Fe in the electrolyte, with the results indicating that higher Fe concentrations led to higher turnover frequency (TOF) values.

The primary focus of this study is to comprehensively investigate the influence of NiO bulk and surface properties on the electrocatalytic performance of ALD NiO films for the OER. By designing these material properties, via two ALD processes, we aim to gain a deeper understanding of their impact on the catalytic performance of ALD NiO films in promoting the OER. Specifically, we address both plasma-assisted and thermal ALD NiO processes, with an emphasis on the extended electrochemical activation process and the OER performance of the films. The results of this study provide new insights and a deeper understanding of the interplay between various material properties and electrocatalytic performance, promoting further engineering of ALD NiO-based electrocatalysts for OER. The forthcoming Results and Discussion section has been strategically structured into distinct subsections, each with a specific focus that collectively contributes to a comprehensive understanding of our research outcomes. The section begins with an in-depth characterization of the as-deposited films based on two pathways, including plasma-assisted and thermal ALD methods. This investigation encompasses a thorough analysis of the film’s chemical, structural, electrical, and morphological properties, all of which influence the electrochemical behavior of the films. Building upon these studies, our investigation transitions to the electrochemical activation. Subsequent subsections examine changes in film chemistry upon activation, followed by an exploration of phase transformations during the activation process. This approach covers the structural changes upon activation and concludes with an investigation of the changes in film morphology, shedding light on the final deactivation of the films and their potential underlying causes.

## Experimental Section

### ALD Synthesis and Sample Preparation

**Scheme 1:**
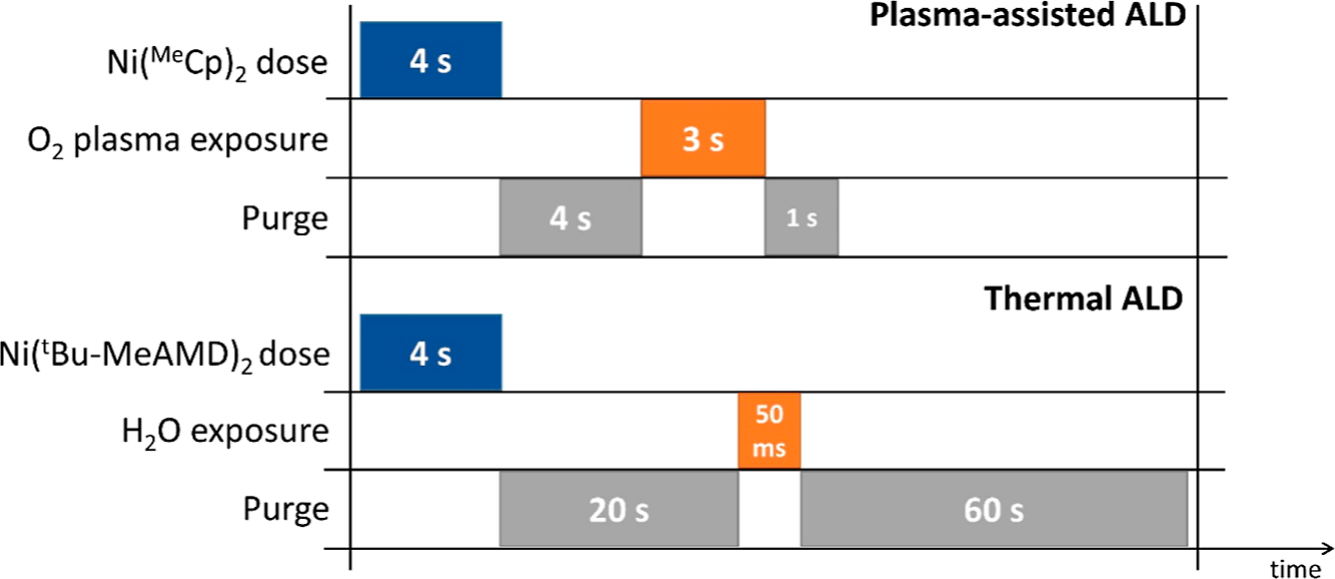
Schematic Illustration of the NiO Film Fabrication Process through ALD

The detailed ALD recipes of plasma-assisted^55^ and thermal ALD^56^ processes have been previously reported, see Scheme 1. In this study, the fabrication of NiO electrocatalysts involved the utilization of a custom-built reactor for the plasma-assisted process and an Oxford Instruments FlexAL reactor for the thermal process. Prior to both ALD procedures, the substrates were cleansed for 15 min with O_2_ plasma to eliminate potential contaminants. In accordance with the process specifications, the growth per cycle (GPC) values for thermal- and plasma-assisted ALD NiO were found to be ∼0.043 and ∼0.030 nm/cycle, respectively. However, the plasma-assisted process is a more time-efficient option for ALD NiO deposition due to its shorter saturation time per cycle. Furthermore, the thermal ALD process was conducted at a growth temperature of 150 °C, whereas the plasma-assisted ALD process involved a higher growth temperature of 300 °C.

In order to examine the electrocatalytic activity, the films were deposited on substrates made of fluorine-doped tin oxide (FTO)–glass (TEC: 10, 20 × 15 × 1.1 mm) purchased from Ossila. FTO has high electrical conductivity, which is crucial for facilitating effective charge transfer during OER. Moreover, FTO has high chemical stability, allowing it to endure the corrosive conditions that often occur throughout OER. Notably, it exhibits negligible activity toward OER, allowing us to analyze the electrocatalytic performance of ALD NiO in more detail.^52,57^ Prior to NiO deposition, FTO-coated glass substrates were sonicated for 10 min in a water/soap solution, acetone, and isopropyl alcohol. During both processes, tiny Si(100) wafers were placed on a fraction of the FTO substrates to serve as a physical mask for connecting the potentiostat for electrochemical analysis. Moreover, Si(100) and highly resistive Si thermal oxide (Si/SiO_2_) substrates were also included in every ALD run for supplemental characterizations.

### Characterization of ALD Films

In situ spectroscopic ellipsometry (SE) using an M-2000U Ellipsometer from J. A. Woollam Company was employed to monitor the film thickness during the ALD process. Using the commercial software CompleteEASE, measurements were carried out on Si wafers, and subsequent data analysis involved the modeling of the collected data using two Tauc-Lorentz oscillators.^58^ X-ray photoelectron spectroscopy (XPS) was conducted using a Thermo Scientific KA1066 spectrometer equipped with a monochromatic Al Kα X-ray source, and samples were examined without any presputtering process. The collected data were evaluated using the licensed program Avantage with Shirley background subtraction. Since the binding energy of the core electrons is impacted by the binding energy of the valence electrons in the sample and causes peaks to shift, the binding energies of the XPS spectra were adjusted by setting the most intense of the carbon peak in the C 1s spectra to 284.8 eV. The high-resolution XPS spectra were analyzed using the fitting parameters for NiO and NiOOH as outlined in the Biesinger et al.^59^ reports. Rutherford backscattering spectrometry (RBS) measurements were carried out using a 1000 keV He^+^ beam with a perpendicular incidence angle to the surface of the sample. Grazing incidence X-ray diffraction (GIXRD) patterns were obtained using an XRD Bruker D8 diffractometer with a Cu Kα (λ = 1.54060 Å) X-ray source. The integration time per step was 14 s, featuring a 0.02° increment, and DIFFRAC.EVA software was used to analyze the patterns. The four-point probe (FPP) measurement was performed on Si thermal oxide (Si/SiO_2_) to guarantee electrical isolation between the ALD NiO film and the substrate. The system was operated by lab-built LabView software and comprised a Signatone probe connected to a Keithley 2400 SourceMeter at room temperature. The vertical resistivity of the films was determined using the current–voltage measurement (four-probe technique) by passing an electric current perpendicular to the surface of the films and obtaining current–voltage curves in which the resistance can then be calculated as the ratio of the voltage to the current. For this approach, the ALD NiO films were deposited on indium–tin-oxide (ITO)-coated glass, followed by a patterned thin layer of silver coating on top, which was applied using an electron beam evaporator (Temescal FC-2000). Lastly, scanning electron microscopy (SEM) images were taken using a field-emission ZEISS Sigma scanning electron microscope. The acceleration voltage was adjusted to 3 kV, and carbon tape was utilized as the conductive coating on both sides to prevent the sample from charging during analysis.

### Electrochemical Measurements

The ALD NiO films were electrochemically activated in a single-compartment, three-electrode electrochemical cell using a CompactStat (Ivium) potentiostat. As the working electrode, NiO samples were loaded into a sample holder with a 1 cm^2^ nominal aperture (Redox.me). In addition, a reversible hydrogen electrode (RHE) (Mini-HydroFlex, Gaskatel) and a graphite rod (Redox.me) were used as the reference and counter electrodes, respectively. It is suggested to employ a graphite rod instead of platinum (Pt) as the counter electrode when examining working electrodes that do not contain platinum group metals (PGMs).^60^ This recommendation is based on the (electro) dissolution and electrodeposition of Pt onto the working electrode, which can cause changes in its surface chemical composition. These modifications have a notable impact on the mechanism and kinetics of the studied process.^61,62^ The activation procedure was carried out in a 1.0 M KOH solution (VWR Chemicals) for 1000 cyclic voltammetry (CV) cycles. The potential range was set between 0.8 and 1.8 V vs RHE, and the curves were obtained at a scan rate of 10 mV s^–1^ for enhanced resolution of oxidation and reduction processes and improved electrode stability. Furthermore, the use of a wide potential range in conjunction with a low scan rate enhances the irreversibility of CV cycling and leads to the accumulation of Ni^3+^ species during activation.^63,64^

The electrochemical impedance spectroscopy (EIS) curves were obtained under identical experimental conditions after 20 CV cycles and at the completion of the activation process. Due to the surface sensitivity of EIS, it was important to consider the potential presence of surface contamination on the electrode prior to conducting the measurements. To mitigate this issue, the films were subjected to 20 sweeps of cycling to remove any contaminants that might have adhered to the electrode interface before the measurement was initiated. The CV plots were corrected using 80% *iR*-compensation, where the Ohmic resistance value was determined by EIS performed at the beginning of the activation process. Following the completion of the experiments, the samples were rinsed for 15 to 20 s with DI water to eliminate any residual electrolyte that may have accumulated at the surface during the measurement and to prepare them for post-CV analysis. The irreversible redox charges were determined by evaluating the integral of the noncatalytic wave area within the potential range of the wave on CV plots. The contribution of the wave observed during the backward scan was then subtracted from that observed during the forward scan. The values calculated represent the total transfer of electric charges (in mC cm_geo_^–2^) during the noncatalytic reaction, which are then converted into the total number of elementary charges transferred (in Ni_atoms_ cm_geo_^–2^). Lastly, the electrochemically active surface area (ECSA) of the films was determined using the adsorbate capacitance (*C*_a_) method.^65,66^ To determine the adsorbate capacitance for the OER in the films, EIS was performed initially after 300 cycles and after 1000 cycles at 1.6 V. Subsequently, the Nyquist plot was fitted with an equivalent circuit model (Figure S1) to obtain the adsorbate capacitance. These obtained capacitance values were then divided by the specific OER adsorbate capacitance (*C*_s_) of NiO to calculate the ECSA of the catalysts.^65^

## Results and Discussion

### Characterization of As-Deposited Films: Chemical, Electrical, and Morphological Properties

**Figure 2:**
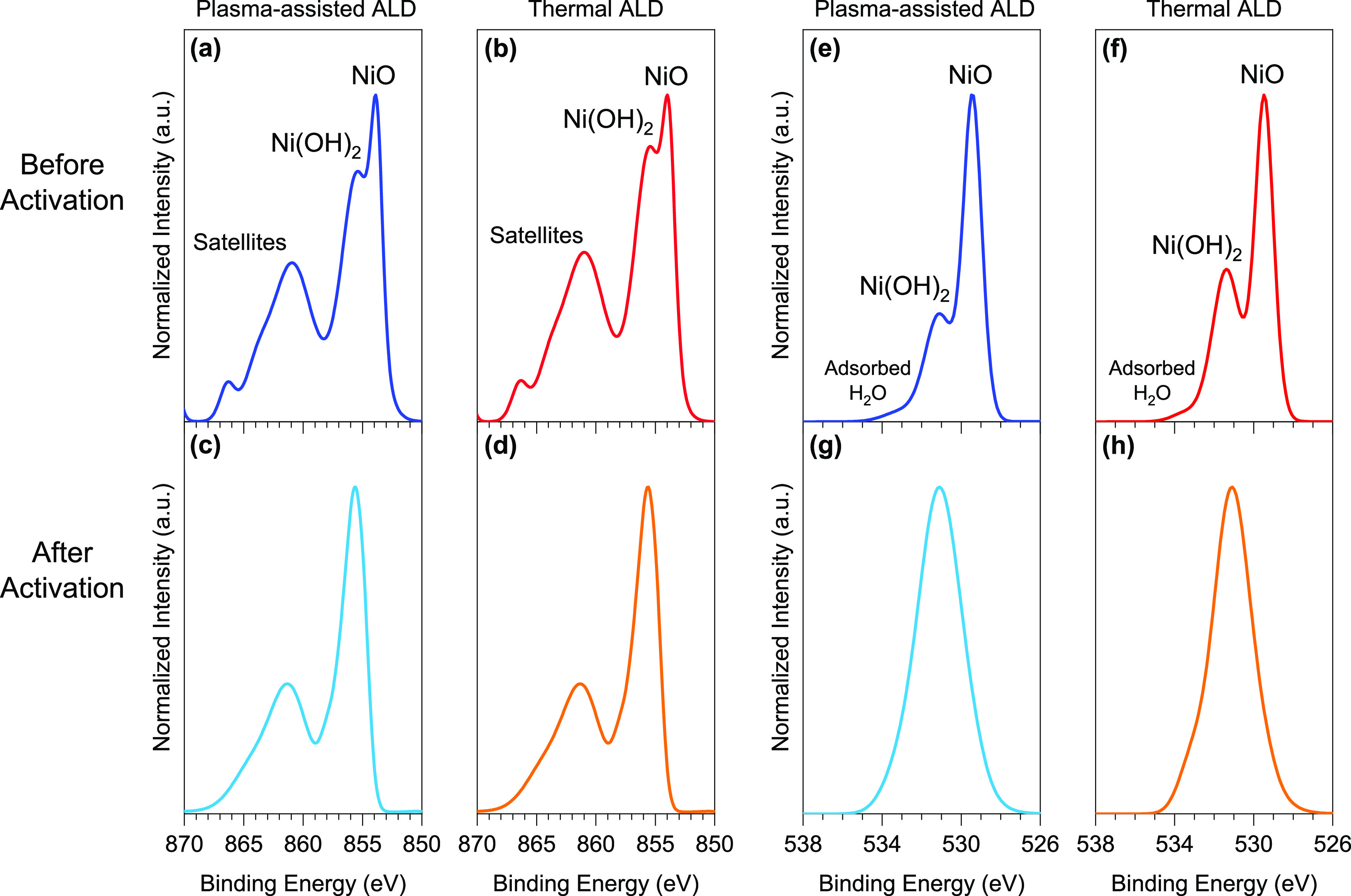
(a–d) Ni 2p and (e–h) O 1s XPS spectra of ∼10 nm ALD NiO films: (a,e) plasma-assisted ALD and (b,f) thermal ALD NiO before activation and (c,g) plasma-assisted ALD and (d,h) thermal ALD NiO after activation.

The surface chemistry of the ALD NiO films deposited on FTO was evaluated by XPS. The survey XPS scans (Figure S2) of both plasma-assisted and thermal ALD NiO samples show that the films are composed of Ni and O at a ratio of 1.13:1.00 of O/Ni, with an estimated error of ±0.04 due to variations in sample preparation, as well as an adventitious carbon layer due to exposure to the ambient environment during sample preparation and handling. The chemical state of the elements can be inferred by high-resolution XPS spectra. The Ni 2p spectrum (Figure S3) is split into 2p_3/2_ and 2p_1/2_ components, owing to significant spin–orbit coupling. It is widely acknowledged that XPS studies for the first row of transition metals and their oxides and hydroxides are challenging. Their 2p spectrum complexity is a result of multiplet splitting, peak asymmetries, overlapping peaks, and satellite features.^59,67,68^ Nevertheless, this XPS study yielded results that are consistent with earlier findings.^55,59,69^ As depicted in Figure 2a,b, the Ni 2p_3/2_ spectra consist of two prominent peaks centered at 853.7 and 855.4 eV, which correspond to NiO and Ni(OH)_2_, respectively, coupled with broad satellite peaks at higher binding energies.^59^ A similar pattern also occurs in the 2p_1/2_ region, where the energy difference between the primary Ni 2p_3/2_ and Ni 2p_1/2_ peaks is 17.5 eV. It is crucial to highlight that we identify the peak centered at 855.4 eV as Ni(OH)_2_ rather than NiOOH. This choice is driven by the potential instability of the NiOOH phase within as-deposited films and the inherent challenge of distinguishing between these two phases using XPS, as their peak centers are in close proximity, with a mere 0.5 eV difference.^70^ The O 1s spectra (Figures 2e,f, and S4) are made up of oxygen atoms bonded as Ni–O–Ni and Ni–OH, as evidenced by XPS features centered at 529.6 and 531.4 eV, respectively, in addition to a contribution from adsorbed H_2_O at higher binding energies.

RBS and elastic recoil detection (ERD) were employed to validate the elemental composition of ALD NiO films deposited on Si(100) and determine the absolute elemental concentration. According to Table 1, the O-to-Ni ratio for plasma-assisted and thermal ALD NiO is 1.03 and 1.06, respectively, which are slightly less than the values estimated by XPS. This overestimation is primarily due to the fact that XPS is a surface-sensitive technique in which the topmost surface dominates the signal, and it is expected that the top surface of NiO will be more oxidized upon exposure to air. Furthermore, it has been observed that the thermal ALD NiO film exhibits a higher hydrogen (H) content compared to plasma-assisted ALD NiO. The presence of hydrogen in the film, such as NiOOH or Ni(OH)_2_, is derived from the chemistry of the coreactant selected for the ALD process. In the thermal ALD process, hydrogen primarily forms through ligand oxidation, while in the plasma-assisted ALD method, both residual moisture in the background and ligand combustion contribute to the presence of hydrogen. This observation is consistent with XPS results, where the intensity of the Ni–OH peak in the O 1s spectrum of thermal ALD NiO is slightly higher than that in the plasma-assisted ALD NiO film. This suggests that a higher amount of intermediate products such as Ni(OH)_2_ and NiOOH is retained during the thermal ALD process.

**Table 1:** Atomic Concentration and Film Mass Density of the ∼15 nm Plasma-Assisted and ∼25 nm Thermal ALD NiO Films^a^

sample	Ni (atoms nm_geo_^−2^)	O (atoms nm_geo_^−2^)	H (atoms nm_geo_^−2^)	O/Ni	density (g cm^–^^3^)
plasma-assisted ALD NiO	786 ± 8	813 ± 32	56 ± 2	1.03 ± 0.06	6.56 ± 0.22
thermal ALD NiO	1009 ± 10	1071 ± 43	262 ± 8	1.06 ± 0.06	5.09 ± 0.17

a RBS and ERD were used to measure atomic densities per unit of geometric surface area. These densities were used to deduce the stoichiometry of the samples, excluding hydrogen due to its negligible contribution.

**Figure 3:**
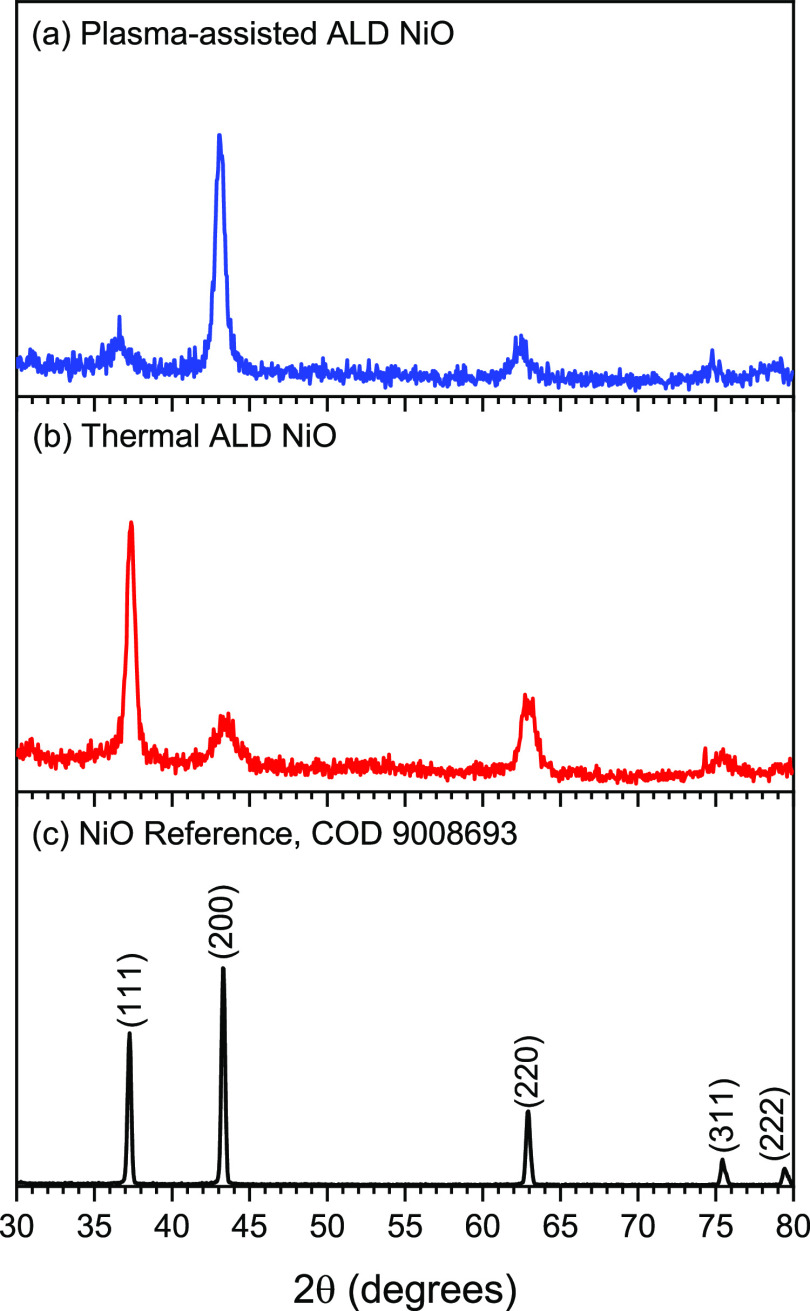
GIXRD patterns of ∼20 nm: (a) plasma-assisted ALD, (b) thermal ALD films on the Si substrate, and (c) reference NiO samples.

GIXRD analysis was employed to identify the crystal structure of samples deposited on Si(100), as shown in Figure 3. The GIXRD patterns of both ALD films are identical to that of the reference pattern, confirming a rock-salt NiO phase. Nevertheless, the two films exhibit different crystallographic features. The patterns indicate that (111) and (200) are the preferred crystallographic orientations for thermal and plasma-assisted ALD NiO samples, respectively. Previous reports suggest that the OER activity of the NiO catalyst is crystallographic-orientation-dependent. The experimental study by Poulain et al.^26^ shows that NiO(111) prepared by sputtering corresponds to a larger hydroxide coverage than NiO(200), resulting in enhanced OER activity. Furthermore, their findings show that both NiO(111) and NiO(200) samples convert into the γ-NiOOH phase upon the activation process, and no phase transformation to β-NiOOH was observed during the electrochemical aging. Besides, Cappus et al.^71^ reported that during the reaction of H_2_O molecules with NiO, hydroxyl groups only bond to defective sites on NiO(200); however, they have a strong interaction with regular sites on NiO(111). Due to the high crystallinity of the FTO substrate, the GIXRD patterns (Figure S5) of both ALD films on FTO exhibit intense diffraction signals caused by the substrate, whereas the NiO peaks are insufficiently intense to be identified. Nevertheless, the plasma-assisted ALD NiO pattern shows the (200) signal as the strongest peak, as there is no corresponding signal from FTO in that region, which demonstrates the growth of the film on the substrate.

To investigate the electrical characteristics of the ALD NiO films, their lateral and vertical resistivities were measured. The lateral resistivity of the films quantifies the ability of electrical charge to flow in the plane of the film, as well as the sideways direction, and was determined by measuring the sheet resistance of the thin films using FPP method. As indicated in Table 2, there is a substantial difference in the lateral resistivity of the films, with plasma-assisted ALD NiO being a factor of 200 more conductive than thermal ALD NiO. The significant variation can be attributed to various factors including impurities, oxygen vacancies, crystal structure, film morphology, and stoichiometry. Nevertheless, the underlying factors responsible for this pronounced contrast in conductivity remain elusive, and further investigation beyond the purpose of this Letter is required to elucidate the precise mechanisms at play.

**Table 2:** Lateral and Vertical Resistivities of the ALD NiO Films

sample	lateral resistivity (Ω cm)	vertical resistivity (Ω cm)
plasma-assisted ALD NiO	68 ± 21	(1.49 ± 0.02) × 10^6^
thermal ALD NiO	(1.70 ± 0.30) × 10^4^	(1.76 ± 0.12) × 10^6^

In parallel, efficient vertical charge transport enables the effective transfer of electrons to and from the electrocatalyst surface during the OER process. Vertical resistivity is generally considered more important, as it directly influences the charge-transfer efficiency through the thickness of the catalyst film, which is a crucial aspect of catalytic activity. As listed in Table 2, the difference in vertical resistivity values compared to the lateral resistivities is relatively minor. This similarity suggests that the conductivity of the films in the vertical direction is comparable, regardless of the different fabrication methods.

**Figure 4:**
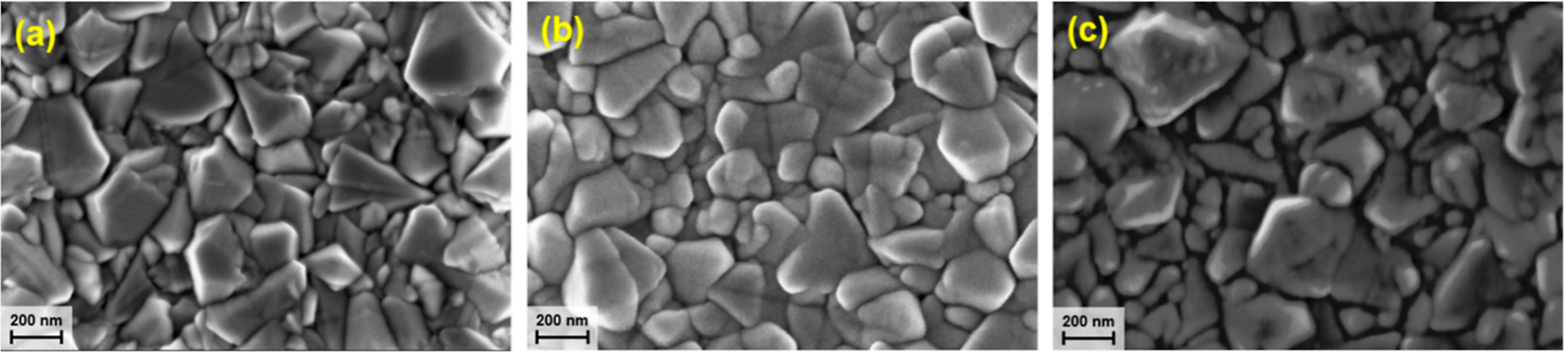
SEM micrographs of (a) pristine FTO substrate and as-deposited ∼10 nm (b) plasma-assisted ALD and (c) thermal ALD NiO films.

To characterize the surface morphology and determine the surface roughness of the electrocatalyst prior to and following electrochemical activation, SEM was employed. Surface roughness and texture can influence the kinetics of charge transfer, the adsorption of reactants and products, and the stability of the electrocatalyst. As illustrated in Figure 4A, the pristine FTO substrate has a rough surface with visible angular and pyramidal shapes. When NiO was deposited (Figure 4B,C), the morphology is retained due to the conformal nature of ALD.

### Electrochemical Activation of NiO Films

**Figure 5:**
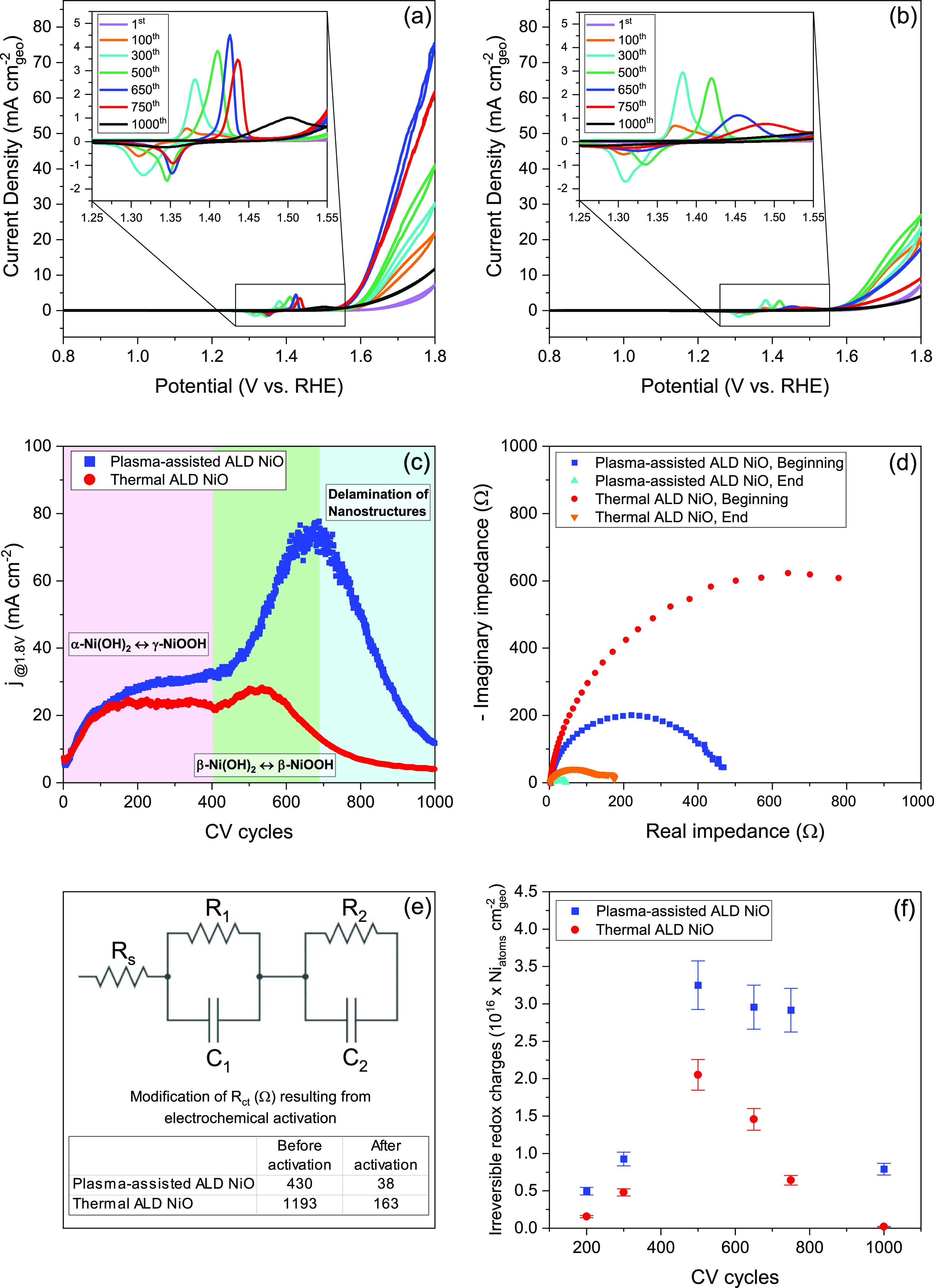
Repeated CV sweeps for the (a) plasma-assisted ALD and (b) thermal ALD NiO films on FTO glass in a 1.0 M KOH solution with a scan rate of 10 mV s^–1^. The inserts report the noncatalytic wave region typical of (oxy)hydroxide phase transformation. (c) Current density (1.8 V vs RHE) as a function of the number of CV cycles. (d) EIS Nyquist plots realized at 1.6 V vs RHE of the ALD NiO films at the beginning and end of electrochemical activation. (e) Equivalent electric circuit model used to fit EIS plots and charge-transfer resistances of the ALD NiO films before and after electrochemical activation. (f) Irreversible redox charges as a function of the number of CV cycles.

In order to evaluate the electrochemical activation process of ALD NiO, up to 1000 CV cycles were carried out, i.e., the number of cycles expected to be sufficient to boost the performance of the electrocatalyst by promoting its activation, either by development of active sites or removal of surface contaminants. Figure 5a,b illustrates the CV curves of plasma-assisted and thermal ALD NiO films at various activation levels. The curves comprise noncatalytic waves in both anodic and cathodic scans located just prior to the OER, revealing the hydroxide/oxyhydroxide phase transformation. In both cases, electrochemical activation enhances the activity of NiO films, as determined by the current density values obtained at 1.8 V vs RHE. Comparing the maximum activity, plasma-assisted ALD NiO outperforms thermal ALD NiO by a factor of 3. The evolution of the electrochemical characteristics of ALD NiO as a function of the number of CV sweeps was carried out, as shown in Figure 5c. In the initial 100 cycles, the performances are comparable with an increase in activity from 6 to 20 mA cm^–2^. In the subsequent 300 cycles, the activity of the thermal ALD film remains steady in the range of 23 ± 2 mA cm^–2^, whereas the activity of the plasma-assisted ALD film increases slightly, approaching 32 mA cm^–2^ after 400 CV sweeps. After the first activation phase, NiO films undergo a second activation process during which the plasma-assisted ALD NiO reaches its maximum activity after ∼700 cycles (77 mA cm^–2^), whereas this step is lacking in the case of the thermal ALD film where the maximum activity is achieved after ∼500 sweeps (28 mA cm^–2^). Upon approaching their maximal activity levels, both films undergo deactivation, with plasma-assisted ALD NiO attaining 12 mA cm^–2^ and thermal ALD NiO reaching 4 mA cm^–2^ after 1000 cycles. Accordingly, the activation process of both plasma-assisted and thermal ALD NiO consists of two activation stages at the beginning and one deactivation phase at the end, which requires more investigation.

To evaluate the changes in the charge-transfer resistance at the interface caused by cycling and activation, EIS analysis was performed prior to and following the activation (Figures 5d and S6). The Ohmic and charge-transfer resistance values were calculated by modeling the interface using the extended Randles circuit^72−74^ and are listed in Figure 5e and Table S1. This circuit was utilized due to the presence of multiple semicircles, particularly following activation. The significant decrease in charge-transfer resistance values following electrochemical activation is due to the formation of more active surface sites at the interface and a more conductive surface layer composed of NiOOH during the activation process. NiOOH has a more ordered and crystalline structure than Ni(OH)_2_, enabling more efficient electron transport within the film. Moreover, the oxygen atoms in NiOOH are more efficiently able to engage in redox reactions than those in Ni(OH)_2_, which also contributes to its enhanced conductivity. The increased oxidation states of nickel in NiOOH compared to those in Ni(OH)_2_ facilitate the engagement of oxygen atoms in reversible redox reactions. This increased redox activity plays a key role in enhancing the conductivity and catalytic efficiency of NiOOH. Furthermore, the lower charge-transfer values in plasma-assisted ALD NiO compared to thermal ALD film imply a reduced resistance to electron transfer and, thus, a faster electron-transfer rate. This indicates that plasma-assisted ALD NiO can transport electrons to the surface of the electrode more effectively, resulting in a greater OER rate, which is in agreement with the findings of CV analysis. The constancy of Ohmic resistance during the activation process indicates that neither the electrolyte nor the electrode–electrolyte interface properties are significantly affected by the electrochemical reactions taking place at the electrode.

Moreover, based on the GIXRD findings from the previous section, it was predicted that the (111) surface, which is the preferred crystallographic orientation in thermal ALD NiO, would exhibit higher OER activity compared to the (200) surface, which is the preferred orientation observed in plasma-assisted ALD NiO. However, the results suggest that other parameters might significantly impact the electrocatalytic activity of the films and may exceed the effect of the favored crystallographic orientation, which requires more exploration.

### Chemical Changes upon Electrochemical Activation

As addressed earlier, the electrochemical activation of the NiO film is always followed by its conversion to NiOOH. Accordingly, the rate of this conversion during the activation process should be quantified since the enhancement of NiOOH concentration results in greater electrocatalytic activity. Therefore, XPS was used to evaluate the changes in the surface chemistry of the activated films after 1000 CV cycles. The survey spectra (Figure S7) indicate the presence of Ni, O, and an adventitious carbon layer, indicating the absence of contamination. In addition, the emergence of the Sn peak following activation, despite its absence in the as-deposited samples, suggests the possibility that the substrate may not have been fully covered by the film after activation, enabling its detection. The Ni 2p_3/2_ spectra (Figures 2c, 3d, and S8) comprise a conspicuous peak at 855.4 eV corresponding to Ni(OH)_2_, along with broad satellite peaks at higher binding energies. In both activated films, the absence of the peak centered at 853.7 eV indicates the full transformation of the NiO layer into Ni(OH)_2_. Although the CV analysis and specifically the observation of the noncatalytic wave clearly point out the transition to NiOOH, ex situ XPS analysis cannot provide further evidence on this conclusion because of the considerations made earlier when discussing Figure 2a and the instability of NiOOH in air, after the electrochemical reactions. In parallel, the O 1s spectra (Figures 2g,h, and S9) also show a rise in hydroxyl characteristics, further supporting the Ni 2p findings.

As stated in the Introduction, an essential factor to assess during the activation process is the uptake of Fe in NiO-based electrocatalysts. Through electrochemical activation processes, NiO exhibits a pronounced affinity for Fe, allowing for efficient Fe uptake within its structure. The origin of this trait is a result of the intrinsic electrochemical properties of NiO, including its capability for redox reactions and a high surface area. Fe is inherently present as an impurity in KOH due to its prevalence in the natural minerals used to manufacture KOH. Since Fe 2p and Ni Auger peaks overlap, it is challenging to study the Fe 2p region. Alternatively, the Fe 3p characteristic at ∼56 eV is investigated, as shown in Figure S10. The lack of a signal in the aforementioned region indicates that Fe is not incorporated during the activation process or that the concentration of adsorbed Fe is below the detection threshold of XPS. Consequently, the impact of Fe impurities on the electrocatalytic activity of the films can be disregarded.

### Structural Evolution during Electrochemical Activation

According to the electrochemical activation mechanism of NiO-based electrocatalysts described in the Introduction, and as XPS suggests a quantitative transformation of both NiO films into Ni(OH)_2_/NiOOH layers, it is essential to investigate the possibility of the formation of different NiOOH polymorphs. Given that the activation process of both films is unaffected by Fe uptake while exhibiting distinct activation characteristics, the hypothesis of the formation of various NiOOH polymorphs is strengthened. Exploring the noncatalytic wave zone in CV curves at various activation levels provides information about the intermediate stages of NiOOH production and the circumstances under which each polymorph forms. For better characterization, the regions are magnified in the CV plots (Figure 5a,b). During the preliminary levels of activation, the electrode surface is not activated and only a few active catalytic sites are available. Therefore, the noncatalytic wave is absent in the first CV sweep and only the capacitive current is observed. After 100 cycles, two anodic peaks centered at ∼1.38 and ∼1.41 V vs RHE start growing. Proceeding the activation process for 300 cycles results in a substantial enhancement in the peak centered at 1.38 V vs RHE and a slight increase in the signal centered at higher potentials. According to previous reports,^75−79^ the formation of different NiOOH polymorphs results in the development of distinct noncatalytic waves, in which the growth of the peak centered at lower potentials in the forward scan is associated with the oxidation of α-Ni(OH)_2_ into γ-NiOOH, while the peak present at a higher potential reflects the transition of β-Ni(OH)_2_ to β-NiOOH. In contrast, the β–β redox feature occurs at lower potentials during the backward scan than the α–γ reaction. This is due to the fact that β-NiOOH is thermodynamically more stable than γ-NiOOH, which is more accessible to anions. The hexagonal structure of β-NiOOH, as opposed to the monoclinic structure of γ-NiOOH, enables a stronger metallic interaction between the nickel and oxygen ions, resulting in a more stable compound.^27,77^ Consequently, the γ-NiOOH phase is the predominant product of the activation process in the early stages of CV measurements. As activation continues, the intensity of the α–γ peak decreases, and the β–β peak begins to rise, in which, after 500 CV sweeps, the contribution of the α–γ peak becomes negligible. Accordingly, during prolonged activation stages, the β-NiOOH phase becomes the major activation product. These modifications in the chemical composition and structure of the catalysts characterize both the first and the second activation phases of the ALD NiO films. During the initial 100 sweeps, the conversion of NiO into NiOOH increases the film activity. According to the irreversible redox charges calculations (Figure 5f), the calculated difference in the number of elementary charges transferred in oxidation and reduction waves is 3.2 and 1.9 times greater in plasma-assisted ALD NiO after 200 and 300 CV sweeps, respectively, than in the thermal ALD film. This indicates that the number of Ni^3+^ cations that are not converted back to Ni^2+^ during each CV cycle is higher in plasma-assisted ALD NiO, indicating a greater generation of NiOOH during the activation procedure. Consequently, the higher production rate of NiOOH in the plasma-assisted ALD NiO film compared to the thermal ALD film is responsible for the better electrocatalytic performance of the plasma-assisted ALD NiO film during the first activation phase. In addition, prolonged activation results in the production of a more OER-active β-NiOOH phase, which is responsible for the second activation stage of both ALD NiO films. This is also in line with the Bode model outlined in the Introduction, which demonstrates that the γ-NiOOH phase transforms into the β-NiOOH phase upon aging. The discrepancy in activation performance during the second activation phase between plasma-assisted and thermal ALD NiO films is also explained by variations in their noncatalytic waves. The β–β oxidation wave approaches its maximum height at the 650^th^ CV cycle in plasma-assisted ALD NiO, but it declines drastically in thermal ALD NiO after the same number of CV cycles. The irreversible redox charge investigations further demonstrate that after 650 CV cycles, the β-NiOOH generation rate is 2.0 times greater in the plasma-assisted ALD NiO than in the thermal ALD film. This is consistent with electrochemical activity plots, which demonstrate that the peak level of activity in plasma-assisted ALD NiO is reached after 650 CV sweeps. Therefore, the superior activity of plasma-assisted ALD NiO at a prolonged number of CV cycles is due to the higher rate of transition from α-Ni(OH)_2_ into β-Ni(OH)_2_ phase, which is 1.5 times faster, resulting in the formation of more β-NiOOH. The findings indicate that the plasma-assisted ALD approach may result in a greater concentration of active sites in the film, promoting the formation of β-NiOOH. It is crucial to highlight that despite the initial focus on vertical resistivities in determining the catalyst’s final activity, the lower lateral resistivity of the plasma-assisted ALD NiO film holds significant potential in enhancing its ability to transport charges and electrons during the activation process and phase transformations. This characteristic might cause more efficient activation of the plasma-assisted ALD film compared to that of its thermal ALD counterpart. Consistent with previous reports, these findings provide further support for the advantageous nature of the β-Ni(OH)_2_/β-NiOOH redox couple in promoting the OER activity. Nevertheless, after a particular range of CV cycles, the noncatalytic wave shifted to higher potentials, and the electrocatalytic activity of both films diminished, requiring additional investigation.

### Evolution of Electrocatalyst Morphology through Electrochemical Activation

**Figure 6:**
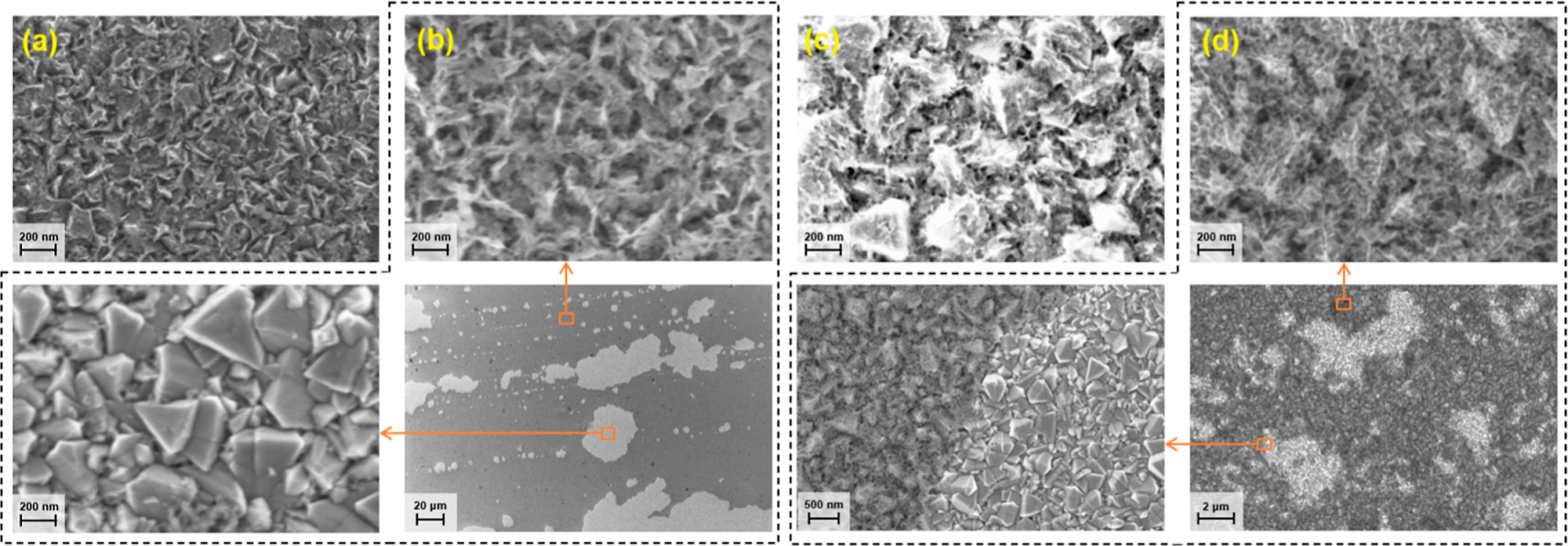
SEM micrographs of plasma-assisted ALD NiO after (a) 300 and (b) 1000 CV sweeps and thermal ALD NiO after (c) 300 and (d) 1000 CV sweeps.

The morphological changes of the films throughout the electrochemical activation process and the primary mechanism for the deactivation of the electrocatalysts during a prolonged activation process were evaluated by employing SEM. The images were collected at two different activation stages: the first stage, after 300 CV sweeps, depicts the morphological changes during the first activation level (Figure 6a,c) and the second, after 1000 CV sweeps, illustrates the second activation and deactivation phases (Figure 6b,d). The SEM images taken after 300 CV sweeps show that the surface morphology of thermal ALD NiO has changed significantly; the pyramidal morphology of the substrate is still discernible, but the surface has a highly textured morphology, indicating the reconstruction of the film after electrochemical activation. Furthermore, the micrograph of plasma-assisted ALD NiO differs considerably from the thermal ALD film, in which the substrate morphology is not identifiable and the structure is rougher, leading to the availability of more active sites. The superior activity of the plasma-assisted ALD NiO film during the second activation phase is primarily attributable to the rougher morphology and greater number of active sites developed during the first activation stage. Notably, the SEM images obtained after 1000 CV cycles indicate two distinct morphologies at various spots on the activated films. The first observed morphology on both films demonstrates a rougher texture compared with the preceding micrographs. The SEM image taken from the thermal ALD NiO film reveals the growth of the previously formed nanostructures, in which the substrate’s pyramidal structure is hardly recognizable. This evolution is also discernible in the image of plasma-assisted ALD NiO, in which prolonged electrochemical activation leads to the vertical growth of nanostructures, resulting in enhanced electrocatalytic activity. In contrast, the second identified morphology has an entirely different structure that is remarkably close to that of the as-deposited films. The enlarged SEM images (Figure 6b,d) of both films reveal that the majority of the films include the morphology described above, possibly developed by the delamination of activated nanostructures. Furthermore, the presence of two semicircles observed in the EIS plots of the activated films (Figure 5d) indicates the existence of two distinct interfaces with varying characteristics and provides evidence supporting the delamination of the activated film. Besides, the presence of Sn in the XPS survey spectrum of the films serves as a confirmation of the separation of activated species, providing further support for morphological modification. Degradation of the activated films also confirms the absence of Fe impurities in electrolytes, which boost the stability of β-NiOOH during the activation of NiO by strengthening its crystal structure and making it more resistant to degradation.^80−83^

**Figure 7:**
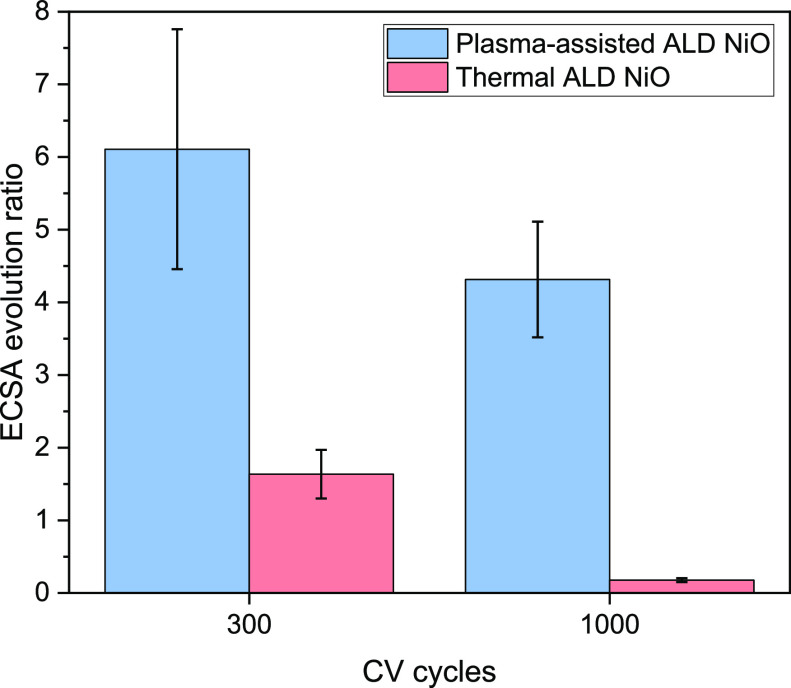
ECSA evolution profiles for ∼10 nm plasma-assisted ALD and thermal ALD NiO films during the electrochemical activation process, showcasing the contrast in the ratios between the ECSA values obtained at the beginning of activation and achieved following 300 and 1000 CV sweeps.

Building upon the SEM characterization, the ECSA analysis provides valuable insights into the interplay between the surface morphology and the electrochemical activity of the ALD NiO samples throughout the activation process. Based on Figure S11, during the initial stage, the plasma-assisted ALD NiO exhibits a slightly lower ECSA value of 0.70 cm^2^ compared to the thermal ALD NiO sample, which shows an ECSA of 0.86 cm^2^. These results indicate differences in the effective surface areas available for electrochemical reactions between the two deposition techniques. The compact nature of the plasma-assisted ALD film may limit the accessibility of active sites, leading to a slightly lower ECSA initially, while the thermal ALD process could provide a relatively larger ECSA due to a potentially more porous surface structure. As the activation proceeds, the ECSA of both samples undergoes notable changes (Figure 7). After 300 cycles, the ECSA of plasma-assisted ALD NiO undergoes a significant boost of 6.1 times, whereas the thermal ALD NiO film exhibits a more modest 1.6-fold increase. This enhancement can be attributed to the progressive formation of active sites and the removal of passivating species on the surface. The plasma-assisted ALD NiO exhibits a greater capacity for electrochemical activity as the increased surface area facilitates efficient charge transfer and promotes the OER kinetics. In contrast, the thermal ALD NiO demonstrates a more limited increase in the ECSA, implying a slower activation process and potentially lower OER efficiency. Remarkably, the obtained ECSA values after 1000 cycles exhibit distinct trends for the plasma-assisted ALD NiO and thermal ALD NiO samples. In the case of plasma-assisted ALD NiO, there is a moderate decrease in the ECSA, resulting in a final ECSA that is 4 times larger compared to the initial value. Conversely, the thermal ALD NiO experiences a substantial reduction in the ECSA, with the final ECSA being 0.2 of the starting value. This decline in the ECSA corresponds to the findings from SEM micrographs, which indicate the detachment of the nanostructured layer from the surface. The delamination process leads to a decrease in the electrochemical activity, contributing to the decreased ECSA value. The mild decrease in ECSA for the plasma-assisted ALD NiO film suggests that the formed nanostructures are rougher compared with those in the thermal ALD NiO film, where partial delamination has a smaller impact on the final surface area. This observation is consistent with the final OER activity of the films, confirming a rougher surface area for the plasma-assisted ALD NiO film. These findings suggest that the plasma-assisted ALD technique offers advantages in terms of maintaining a higher ECSA throughout the cycling process, indicating greater potential for prolonged electrochemical activity. However, the deactivation and eventual decrease in ECSA highlight the challenges associated with maintaining stable nanostructures on the surface.

We ascribe the degradation of the activated films to several potential mechanisms. During the activation process, the development of a passivation layer on the electrode surface serves as a barrier that hinders the flow of electrons and ions between the electrode and the electrolyte, leading to the degradation of the activated film.^84^ The enhanced roughness and number of active sites result in a faster rate of electrolyte penetration and electrochemical reactions, a quicker formation of the passivation layer on the electrode surface, and a faster delamination of the active species, leading to surface degradation. Another probable reason is the overoxidation of the NiOOH layer because of the extended activation process and the production of Ni^4+^ characteristics, which results in the deactivation of the catalyst.^78,85,86^ Particularly, the shift of the noncatalytic wave to higher potential ranges suggests the formation of Ni-based compounds with a greater oxidation state. Once subjected to an ambient environment, Ni^4+^ potentially reacts with water vapor or oxygen to generate NiOOH or Ni(OH)_2_, which are more stable compounds than Ni^4+^. Therefore, the detection of Ni^4+^ by using surface-sensitive techniques, particularly XPS, is challenging.

## Conclusions

To summarize, the electrocatalytic activity of plasma-assisted and thermal ALD-prepared NiO films toward the OER in alkaline media was investigated. Employing ALD offers benefits such as precise control over the thickness and composition of the electrocatalyst layer as well as high uniformity of the film, all of which have a significant impact on the performance of these electrocatalysts. Characterization of the as-deposited NiO films indicates that employing different ALD processes can effectively alter the film characteristics and create NiO films with distinctive properties. Notably, GIXRD studies demonstrated that two films have different crystallographic characteristics, with (111) and (200) being the preferred orientations for thermal and plasma-assisted ALD NiO samples, respectively, given that NiO(111) is more active than NiO(200). Nevertheless, the findings indicate that other factors, such as the enhanced capability for phase transformation during activation and the specific surface area of the films, have a substantial influence on the electrocatalytic activity of the films and outweigh the effect of the preferred crystallographic orientation.

The electrochemical activation process for ALD NiO films begins with two activation phases and ends with one deactivation step. During the activation phases, the formation of a more OER-active NiOOH layer increases the film activity. The noncatalytic wave studies indicate that γ-NiOOH is the product of the first activation phase, during which the formation level in plasma-assisted ALD NiO is larger, resulting in a film with 40% greater activity than thermal ALD NiO. Because of the aging of the activated films, a more OER-active β-NiOOH phase is generated in the second activation phase. The formation of β-NiOOH is significantly enhanced in plasma-assisted ALD NiO compared to thermal ALD NiO, leading to a 3-fold increase in activity. In contrast, the growth of this phase is limited in the case of thermal ALD NiO. The improved ability of plasma-assisted ALD NiO to generate β-NiOOH can be attributed to its lower lateral resistivity. This characteristic holds great promise for enhancing its capacity to facilitate charge and electron transport during the activation process and phase transformations. Moreover, the 3-fold increase in roughness during the initial activation phase of plasma-assisted ALD NiO leads to a larger ECSA, resulting in a significant and sustained activity that persists until the deactivation step. According to morphological studies, the gradual deactivation of the films and a decline in their activity at the end of the activation process are caused by delamination of the nanostructured layer during prolonged activation stages.

## Supporting Information Available

Schematic representation of the equivalent electric circuit for determining the adsorbate capacitance, XPS survey spectra of as-deposited and activated plasma-assisted ALD and thermal ALD NiO films, fitted XPS Ni 2p spectra of as-deposited and activated plasma-assisted ALD and thermal ALD NiO films, grazing-incidence XRD patterns of plasma-assisted ALD and thermal ALD NiO on FTO and pristine FTO–glass, XPS Fe 3p spectra of the ALD NiO films after electrochemical activation, and comparison of ECSA evolution for ∼10 nm plasma-assisted ALD and thermal ALD NiO films during activation (PDF)
