# Supplementary material for: Electrochemical Activation of Atomic-Layer-Deposited Nickel Oxide for Water Oxidation
Source: J Phys Chem C Nanomater Interfaces. 2023 Nov 8;127(46):22570–82. doi: 10.1021/acs.jpcc.3c05002 (PMC10683065; doi:10.1021/acs.jpcc.3c05002)
Supplement: Supplementary file 1 — jp3c05002_si_001.pdf [file jp3c05002_si_001.pdf]

Electrochemical Activation of Atomic Layer Deposited Nickel Oxide for Water Oxidation

Sina Haghverdi Khamene[1,2], Cristian van Helvoirt[1], Mihalis N. Tsampas[2], Mariadriana Creatore[1,3]*

[1] Department of Applied Physics and Science Education, Eindhoven University of Technology, 5600 MB Eindhoven, The Netherlands

[2] DIFFER - Dutch Institute For Fundamental Energy Research, 5612 AJ Eindhoven, The Netherlands

[3] Eindhoven Institute for Renewable Energy Systems (EIRES), 5600 MB Eindhoven, The Netherlands

*E-mail: s.haghverdi.khamene@tue.nl

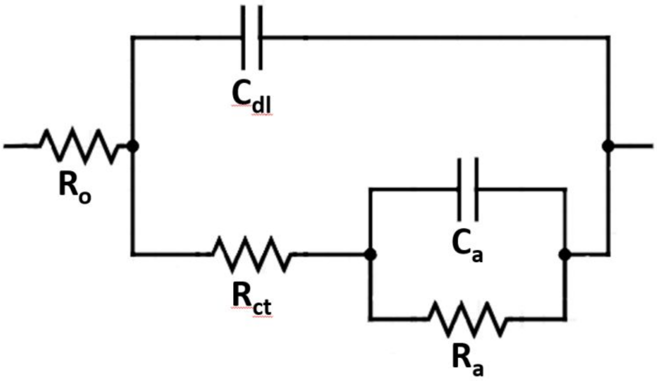

Figure S1. Schematic representation of the equivalent electric circuit for determining the adsorbate capacitance (C_a).

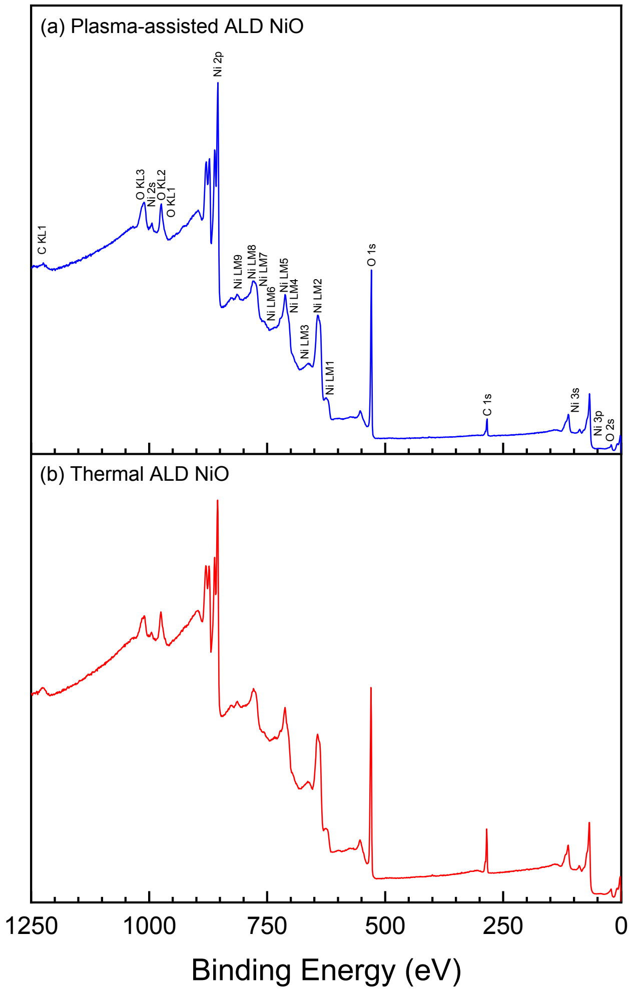

Figure S2. XPS survey spectra of as-deposited (a) plasma-assisted ALD and (b) thermal ALD NiO.

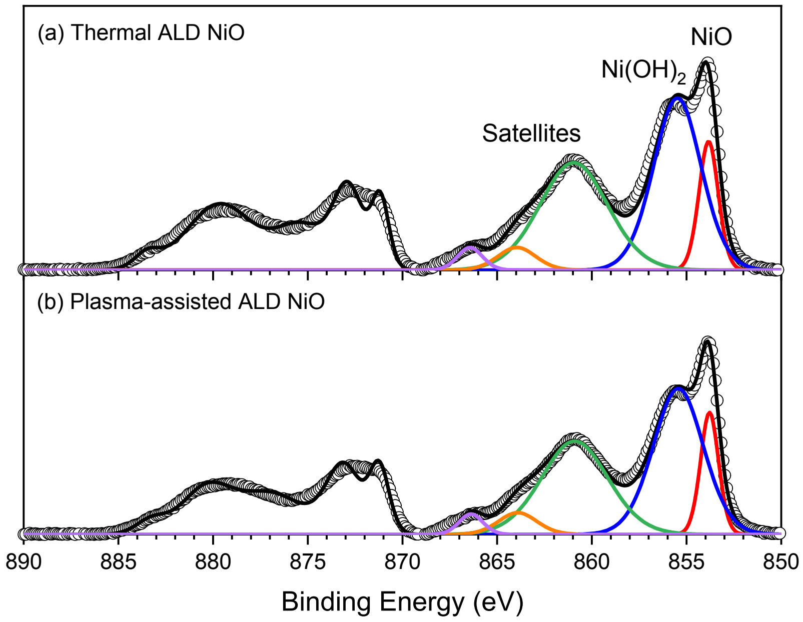

Figure S3. XPS Ni 2p spectra of as-deposited (a) thermal ALD and (b) plasma-assisted ALD NiO.

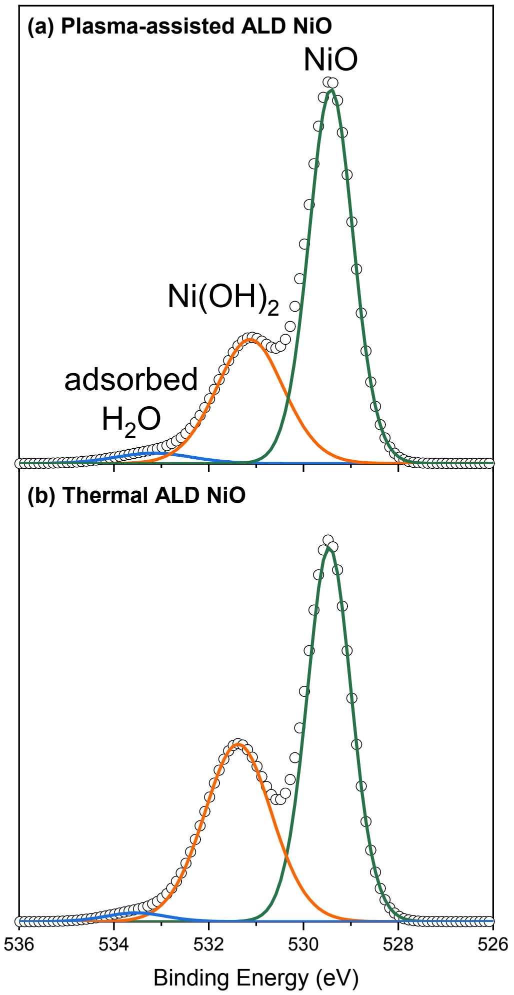

Figure S4. XPS O 1s spectra of as-deposited (a) plasma-assisted ALD and (b) thermal ALD NiO.

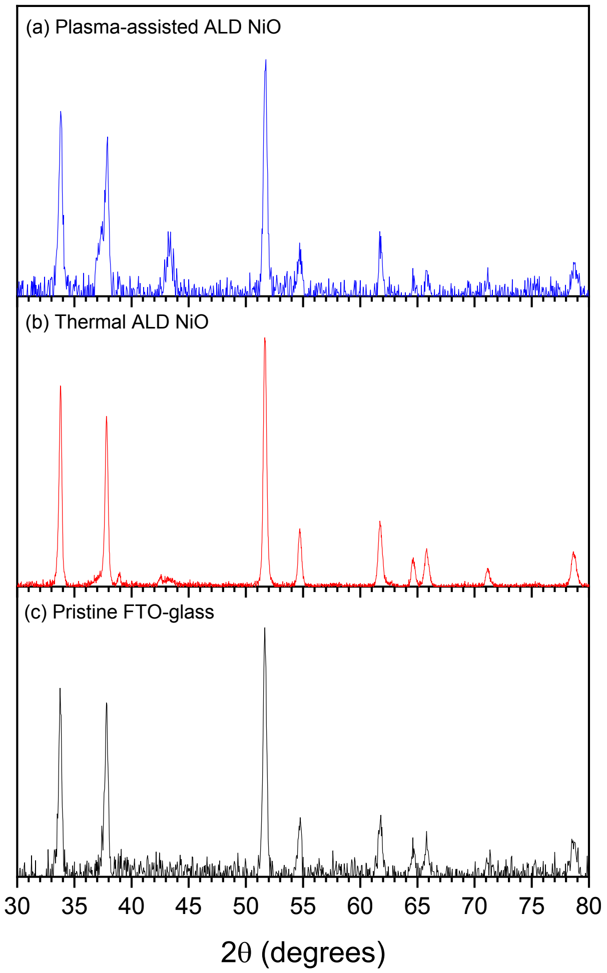

Figure S5. Grazing-incidence XRD patterns of (a) plasma-assisted ALD and (b) thermal ALD NiO on FTO, and (c) pristine FTO-glass.

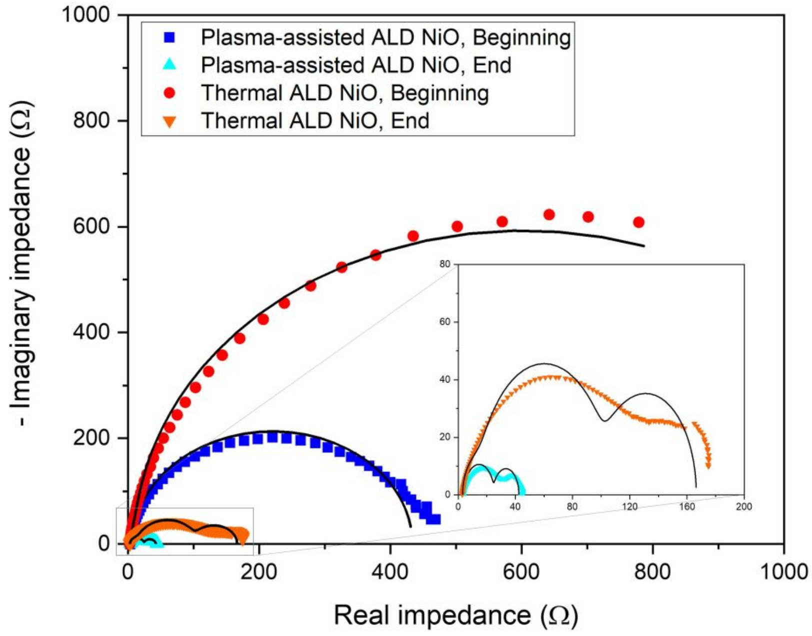

Figure S6. Fitted EIS plots of plasma-assisted and thermal ALD NiO at the beginning and end of activation. The inset highlights the higher-resolution EIS plots at the end of the activation process.

Table S1. Equivalent circuit model parameters extracted from EIS fitting.

Sample		R1 (Ω)	R2 (Ω)	R3 (Ω)	C1 (mF)	C2 (mF)
Plasma-assisted ALD NiO	Beginning	3.46±0.02	425.70±3.49	4.25±0.67	0.288±0.003	1.37±0.10
	End	4.01±0.10	17.71±0.90	20.76±0.56	0.58±0.07	0.006±0.000
Thermal ALD NiO	Beginning	2.80±0.01	1185±20.38	7.91±0.86	0.98±0.08	3.6±0.17
	End	3.05±0.05	64.85±2.98	98.55±2.89	0.97±0.10	0.040±0.002

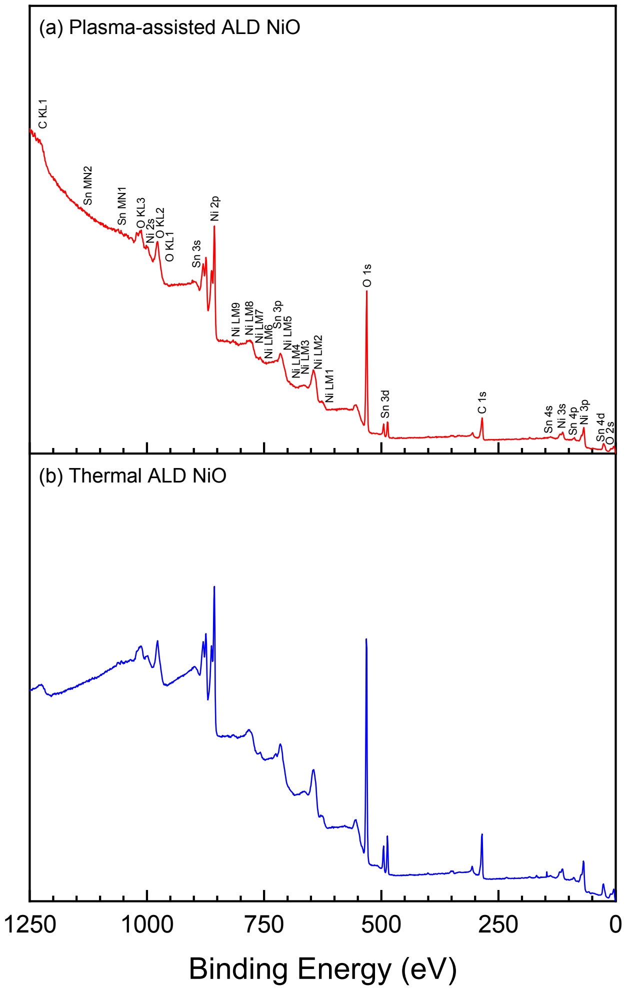

Figure S7. XPS survey spectra of (a) plasma-assisted ALD and (b) thermal ALD NiO films after activation.

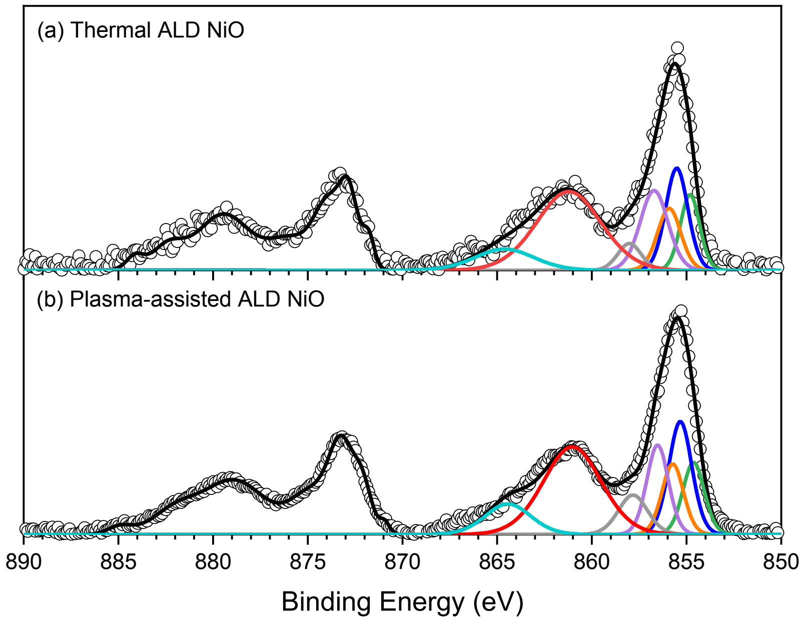

Figure S8. XPS Ni 2p spectra of (a) thermal ALD and (b) plasma-assisted ALD NiO after activation.

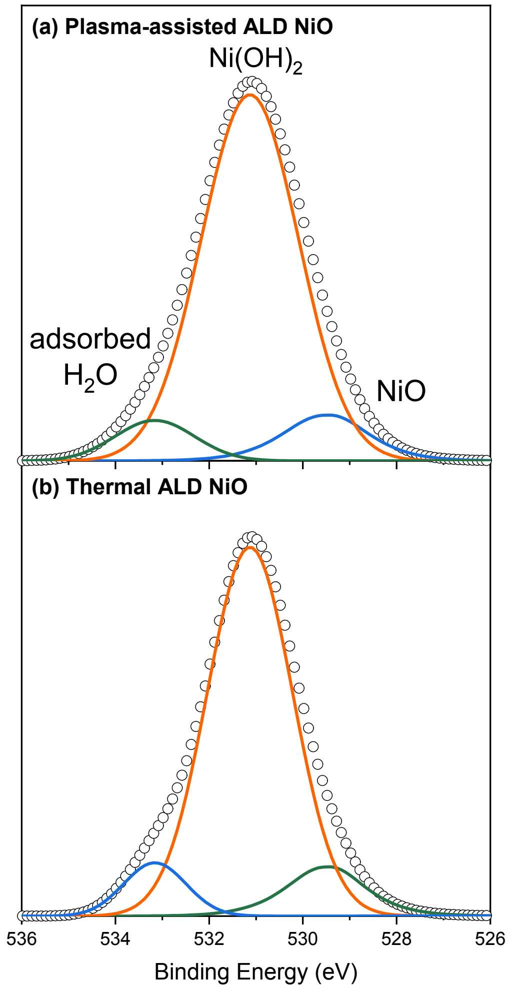

Figure S9. XPS O 1s spectra of (a) plasma-assisted ALD and (b) thermal ALD NiO after activation.

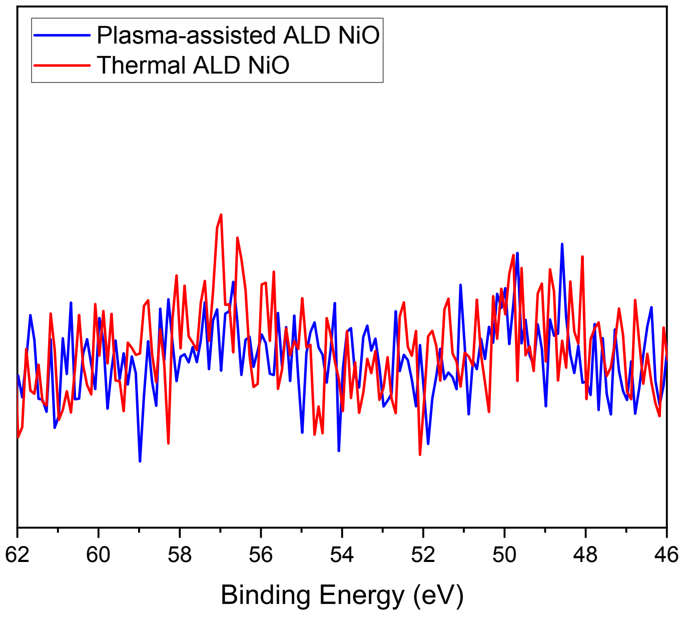

Figure S10. XPS Fe 3p spectra of the ALD NiO films after activation.

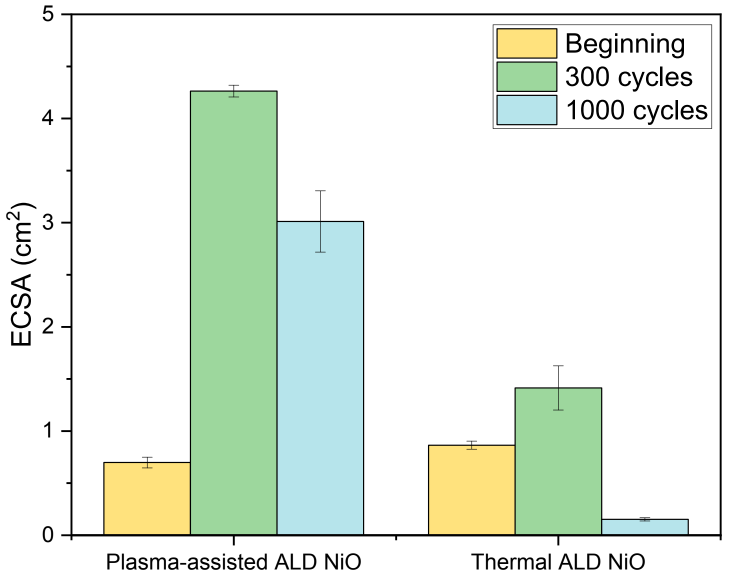

Figure S11. Comparison of ECSA evolution for ~10 nm plasma-assisted ALD NiO and thermal ALD NiO films during activation.

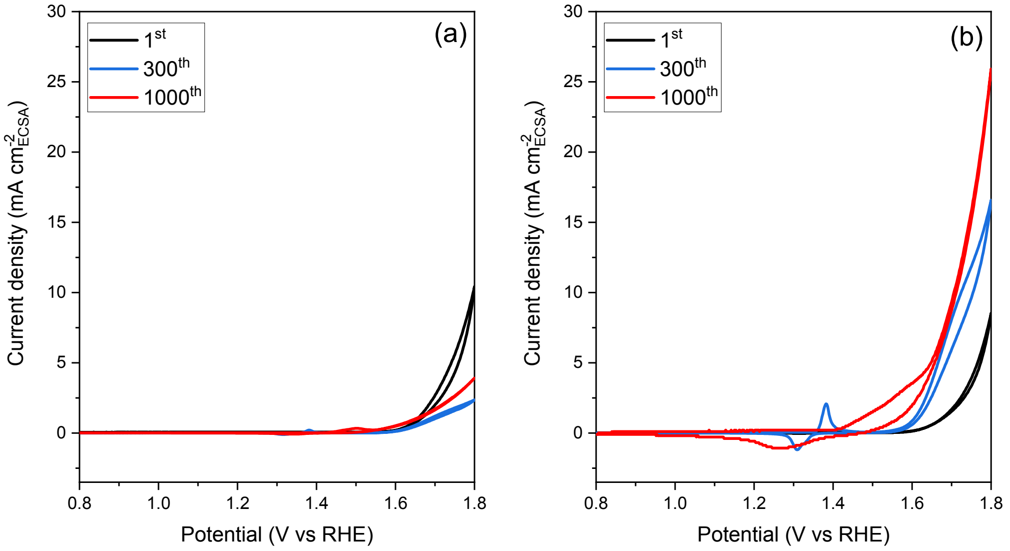

Figure S12. ECSA-corrected CV plots of (a) plasma-assisted ALD and (b) thermal ALD NiO films on FTO-glass in 1.0 M KOH solution with a scan rate of 10 mV·s^{-1}.

Figure S12 displays the ECSA-corrected CV curves for both plasma-assisted and thermal ALD NiO films as a point of reference. In our study, it's important to note that ECSA alone cannot serve as the sole determining factor. This is because we conducted a thorough examination of two distinct ALD NiO films, each possessing its own unique characteristics. Moreover, the electrochemical activation induces modifications in the film's chemistry and structure. Consequently, the nature of the active sites differs between the two films, leading us to the conclusion that a higher concentration of active sites in a sample does not necessarily correspond to a greater OER activity. This variation in intrinsic activity among the sites implies that normalizing CV plots by ECSA could potentially lead to misleading conclusions in our particular case.
